# Mutation analysis for newly suggested 30 Y-STR loci with high mutation rates in Chinese father-son pairs

**DOI:** 10.1038/s41598-022-20014-z

**Published:** 2022-09-20

**Authors:** Fei Wang, Feng Song, Xindi Wang, Mengyuan Song, Yuxiang Zhou, Jing Liu, Zheng Wang, Yiping Hou

**Affiliations:** 1 Institute of Forensic Medicine, West China School of Basic Medical Sciences and Forensic Medicine, Sichuan University, Chengdu, 610041 Sichuan Province People’s Republic of China; 2 Department of Laboratory Medicine, West China Hospital, Sichuan University, Chengdu, 610041 Sichuan Province People’s Republic of China; 3 Med+Molecular Diagnostics Institute of West China Hospital/West China School of Medicine, Chengdu, 610041 Sichuan Province People’s Republic of China

**Keywords:** Genetic markers, Evolutionary genetics, Genetics, Haplotypes

## Abstract

Rapidly mutating Y-STRs (RM Y-STRs) harbor great potential to distinguish male relatives and achieve male identification. However, forensic applications were greatly limited by the small number of the initially identified 14 RM Y-STRs. Recently, with the emergence of 12 novel RM Y-STRs, an integrated panel named RMplex was introduced, which contains all 26 RM Y-STRs and four fast mutating Y-STRs (FM Y-STRs). To obtain the first data on the mutation rates and father-son differentiation rates of the 30 newly proposed Y-STRs in Chinese populations, we performed an empirical mutation study on 307 DNA-confirmed Chinese paternal pairs. Previously reported mutation rates for 14 RM Y-STRs in Chinese and European populations were pooled and merged with our data. The highest meiosis number for the two groups reached 4771 and 2687, respectively. Five loci showed significant differences between the populations (DYS570, DYS399S1, DYS547, DYS612, and DYF403S1b). For the new panel covering 30 Y-STR loci, our results show extensive differences in the mutation rates between the two populations, as well. 10 RM Y-STR loci showed relatively low mutation rates (10^–3^–10^–2^ per meiosis) and 2 FM Y-STR loci had rapid mutation rates (> 10^–2^ per meiosis) in the Chinese population. Several-fold differences in mutation rates were found in nine Y-STR loci between the Chinese and reference populations, with two loci having significantly higher mutation rates and one locus with a significantly lower mutation rate in the Chinese population (*P* < 0.05). Eighteen RM Y-STRs (> 10^–2^ per meiosis), 8 FM Y-STR loci (5×10^–3^-10^–2^ per meiosis), 3 moderately mutating Y-STRs (MM Y-STRs, 10^–3^-5×10^–3^ per meiosis), and one locus with no observed mutation events were identified in the Chinese population. 40.06% of the Chinese paternity pairs were discriminated with RMplex while only 20.84% with the initial 14 RM Y-STRs, indicating that RMplex is beneficial for distinguishing paternally related males. Future studies on populations of different genetic backgrounds are necessary to obtain comprehensive estimates of mutation rates at these new loci.

## Introduction

In forensic genetics, the non-recombining part of the Y chromosome (NRY) has particular advantages in differentiating male components from mixed stains, tracing paternal lineage, and investigating population admixture history, where autosomal DNA profiling is ineffective^1–5^. Since their discovery in 1990, Y-chromosomal short tandem repeats (Y-STRs) have been used to generate unique haplotypes of different paternal pedigrees due to high overall mutation rate (10^–3^ mutations per meiosis)^6,7^. However, the male-limited inheritance and the non-recombining characteristic suggest that the DNA profiling of general Y-STRs usually points to a male perpetrator and his many paternally related relatives, sometimes even unrelated men, which limits the forensic applications and weakens the validity of evidence in court^8^. Therefore, Y-chromosome genetic markers with high mutation rates are essential for identifying male individuals from the same paternal line.

In 2010, a large empirical study on Y-STRs mutation rate analysis introduced 14 RM Y-STRs (including DYF387S1, DYF399S1, DYF403S1a/b, DYF404S1, DYS449, DYS518, DYS526a/b, DYS547, DYS570, DYS576, DYS612, DYS626, and DYS627, with DYF403S1a and DYF403S1b considered as separate loci) whose mutation rates > 10^–2^ per meiosis, providing a powerful tool for distinguishing patrilineal male relatives^9^. Over the last decade, a series of studies on both related and unrelated men has demonstrated the applicability of the 14 RM Y-STRs in forensic genetics^10–16^. However, the relatively small number of RM Y-STRs limited the ability to discriminate between male relatives. A study based on hundreds of father-son pairs showed that 14 RM Y-STRs could distinguish 27%, 46%, 54%, and 62% of male relatives separated by 1, 2, 3, and 4 meiotic divisions, respectively, which is insufficient for forensic applications^10^.

Recently, to overcome this dilemma, an empirical study based on 1616 DNA-confirmed father-son pairs identified 12 novel RM Y-STRs^17^. Subsequently, an integrated panel named RMplex, containing all 26 RM Y-STRs and four FM Y-STRs, was introduced into forensic genetics^18^. The availability of these novel markers with high mutation rates provided a more powerful tool to distinguish paternally related males. The integrated system further facilitated the forensic investigation of large populations, as well. Based on the RMplex, Neuhuber et al. empirically studied 530 Austrian father-son pairs and found that the mutation rates at these loci were largely in consistence with those in the European population in the original paper^19^.However, there is still no data on the father-son differentiation rate or the mutation rate of the new RM Y-STRs in Chinese populations.

Herein, for the first time, we investigated 30 Y-STRs with high mutation rates in 307 Chinese father-son pairs using the RMplex panel. The purpose of this study was to estimate the mutation rates of these novel loci in a Chinese population and to investigate their paternal differentiation rates in order to establish an experimental basis for addressing forensic practice in this population. In addition, we collected and compared mutation rate data from the Chinese population with those from the European reference populations to explore genetic differences between the inter-continental populations.

## Results and discussion

### Mutation rates obtained in Chinese population

A total of 169 mutation events were observed in 9202 allelic transfers, with an average mutation rate of 18.4 × 10^–3^ per meiosis (95% CI 15.7–21.3 × 10^–3^). For each locus, 93.49% of the events belonged to one-step mutations and the rest to two-step and three-step mutations (Table 1), which is consistent with the findings in previous papers^9,17^. The locus mutation rate ranged from < 3.26 × 10^–3^ (95% CI 0.0–11.9 × 10^–3^) for DYS1003 to 65.15 × 10^–3^ (95% CI 40.2–98.8 × 10^–3^) for DYF1001. In the mutation event, the gains and losses were 89 and 80, respectively, with no significant bias, which is also consistent with previous studies^20^. The number of pairs distinguished by 1, 2, 3, and 4 mutation events was 92, 22, 6, and 3, respectively, demonstrating the active mutational potential of these loci, in agreement with previous studies^9,17^.

**Table 1:** Mutation information of the 30 Y-STRs in Chinese Han population.

Locus	N	Mutation rate(× 10^−3^)	95% CI(× 10^−3^)	Number of gains	Number of losses	Number of one-step mutations	Number of two-step mutations	Number of three-step mutations
DYF393S1	2	6.51	[0.8,23.3]	0	2	2	0	0
DYS627	4	13.03	[3.6,33.0]	3	1	4	0	0
DYS570	1	3.26	[0.1,18.0]	0	1	1	0	0
DYS713	2	6.51	[0.8,23.3]	1	1	2	0	0
DYS526b	5	16.61	[5.4,38.3]	4	1	5	0	0
DYF1000	7	22.80	[9.2,46.4]	2	5	6	1	0
DYS518	2	6.51	[0.8,23.3]	0	2	2	0	0
DYS1003	0	< 3.26	[0,11.9]	0	0	0	0	0
DYS1012	3	9.84	[2.0,28.5]	2	1	2	0	1
DYS1005	6	19.54	[7.2,42.0]	1	5	6	0	0
DYS1010	4	13.03	[3.6,33.0]	0	4	4	0	0
DYS1007	3	9.77	[2.0,28.3]	2	1	3	0	0
DYR88	8	26.06	[11.3,50.7]	5	3	7	1	0
DYF404S1	5	16.29	[5.3,37.6]	2	3	5	0	0
DYF387S1	13	42.35	[22.7,71.3]	8	5	13	0	0
DYS1013	5	16.29	[5.3,37.6]	0	5	5	0	0
DYS712	16	52.12	[30.1,83.3]	8	8	15	1	0
DYS711	5	16.29	[5.3,37.6]	3	2	4	1	0
DYS626	3	9.77	[2.0,28.3]	3	0	2	1	0
DYF399S1	15	48.86	[27.6,79.3]	7	8	14	1	0
DYS449	1	3.26	[0.1,18.0]	0	1	1	0	0
DYS724	11	35.83	[18.0,63.2]	7	4	11	0	0
DYS547	4	13.03	[3.6,33.0]	1	3	3	1	0
DYS576	6	19.54	[7.2,42.0]	4	2	4	2	0
DYS612	2	6.51	[0.8,23.3]	1	1	2	0	0
DYF1002	5	16.29	[5.3,37.6]	4	1	5	0	0
DYF1001	20	65.15	[40.2,98.8]	15	5	19	1	0
DYF403S1a	7	22.80	[9.2,46.4]	5	2	7	0	0
DYS442	1	3.26	[0.1,18.0]	1	0	1	0	0
DYF403S1b	3	9.77	[2.0,28.3]	0	3	3	0	0

N: Number of mutation events.

### Haplotype mutations with the RMplex

During the amplification of the first assay of RMplex system, a number of dropouts occurred at specific loci. There were eight dropouts (six at DYS526b and two at DYS1012), and the father-son pairs showed the same haplotype, suggesting possible segmental loss on the Y chromosome or sequence mutations in the primer binding region. Allelic loss was also observed at the DYF1001 locus, with a shift from 71, 81.2 to 71 in a father-son (Supplementary Table S1). Notably, seven multi-copy loci showed expansions of copy number in some father-son pairs (DYS1012, DYF1000, DYR88, DYF387S1, DYF399S1, DYS724, and DYF1001). For example, X155C and X155F showed a five-copy genotype at DYS1000 (reported as a four-copy locus), 3-copy genotype s for DYR88 and DYF387S1 (reported as two-copy loci), and 4-copy genotype s for DYF399S1 and DYF1001 (reported as three-copy loci) by Ralf et al^17^. Duplications of DYF387S1 have also been reported previously to be associated with haplogroup-specific ancestral Y chromosome mutations and structural rearrangements in the azoospermia factor c region^21,22^. A two-step expansion of copy number was observed at DYF1001 (272F and 272C), who showed a five-copy haplotype at DYF1001, a three-copy locus as described in the previous studies. These mutations suggested the possible occurrence of segmental duplications on the Y chromosome of the Chinese group.

**Figure 1:**
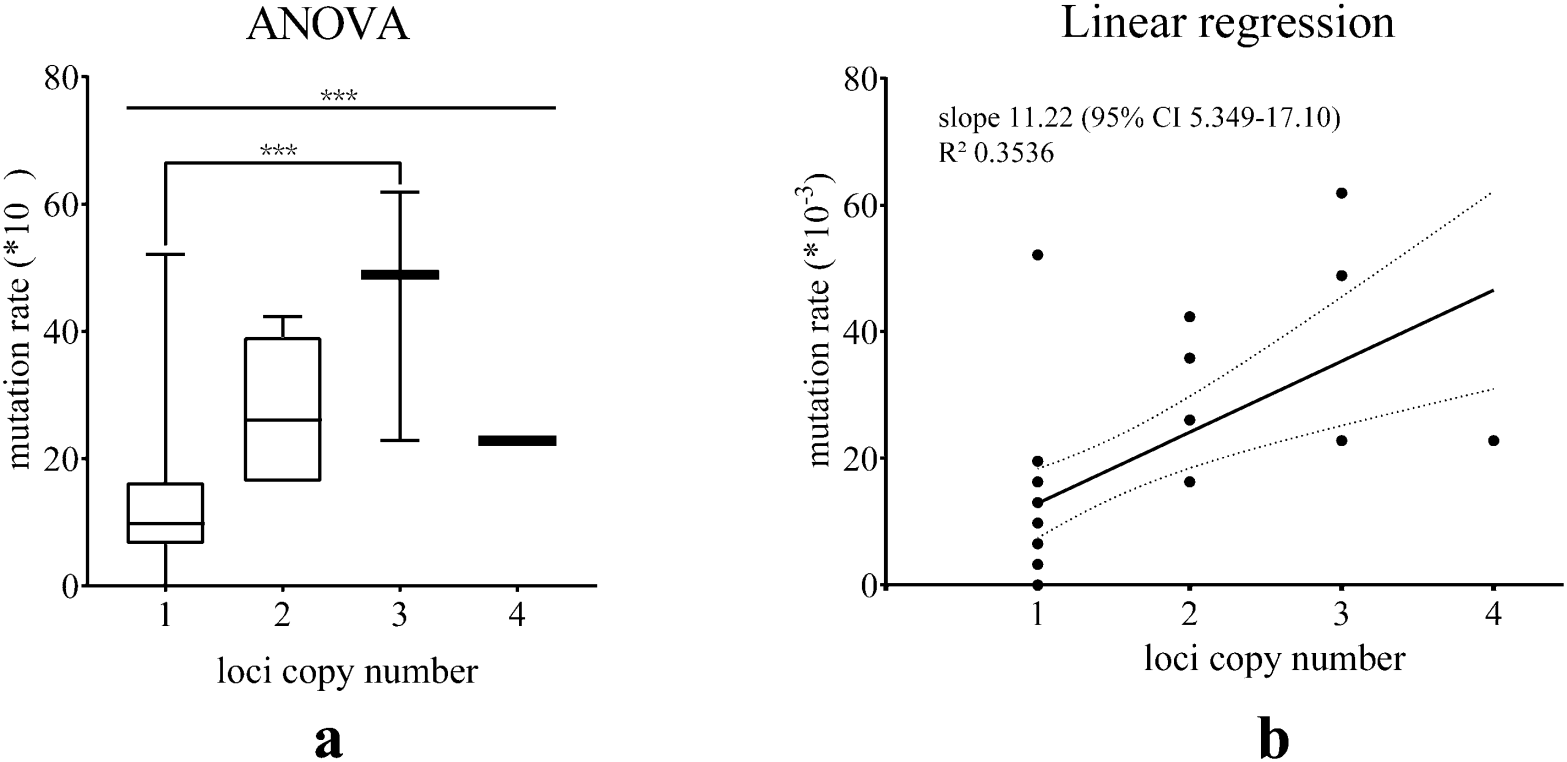
(**a**) One-way ANOVA for mutation rates on loci with different copy numbers; (**b**) Linear regression analysis for mutation rates on loci with different copy numbers (dashed lines represented the 95% CI).

It has also been noted that the mutation rate of the locus rises with increasing copy number. A one-way ANOVA (Fig. 1a) was performed to test whether there was a significant difference in mutation rates of the loci with different copy numbers. Significant differences in mutation rates were observed in all groups (*P* value 0.0006) and between single copy loci and triple copy loci (*P* value 0.0007). To quantitatively assess the correlation between mutation rate and copy number, we conducted a linear regression analysis. The elevated trend was pronounced, although some outliers weakened the centrality of data points, which may be related to stochastic effects due to the small sample size. Notably, that among the single copy loci, DYS712 exceeded the group, exhibiting a relatively high mutation rate of 52.12 × 10^–3^ per meiosis, significantly larger than the mutation rate data obtained from the original study in the German and Polish populations (27.2 × 10^–3^ per meiosis, *P* value 0.0301, Table S2). Overall, the correlation between copy number and mutation rate indicating that each copy of multi-copy loci can be involved in the mutation process as a relatively independent unit, which is in line with the speculation proposed in the previous study^18^.

### Inter-population genetic differences in mutation rates

To assess the differences in the mutation rates of the 14 RM Y-STR between different populations, we combined the obtained data with all previously published data on Chinese^13,15,23–26^ and European populations^9,18,19,27^ (Table 2, Supplementary Table S5, Supplementary Figure S1). For the combined Chinese population, the number of meioses per locus ranged from 3605 (DYS526b) to 4771 (DYS627, DYS570, DYS518, DYF387S1, DYS449 and DYS576). The average mutation rate ranged from 6.50 × 10^–3^ (95% CI 4.4–9.2 × 10^–3^) at DYS570 to 54.83 × 10^–3^ (95% CI 47.6–62.8 × 10^–3^) at DYS399S1, which was the same as the lowest and highest mutation rates among the 14 RM Y-STRs in our group. For pooled European populations, the underlying meioses ranged from 2529(DYS626) to 2687(DYS627, DYS518, DYF387S1, DYF399S1, DYS449, DYS576, DYS612, and DYF403S1a). The mutation rates ranged 10.4 × 10^–3^ (95% CI 6.7–15.0 × 10^–3^) at DYS626 to 78.4 × 10^–3^ (95% CI 68.6–89.3 × 10^–3^) at DYF399S1. Fisher's exact test for mutation rates between the two populations for each of the 14 RM Y-STR loci revealed significant differences at five of the loci (DYS570, DYS399S1, DYS547, DYS612 and DYF403S1b). The wide range of differences observed suggests that there are non-negligible differences in mutation between different continental groups.

**Table 2:** The mutation rates of 14 RM Y-STRs in combined Chinese and European populations.

Combined Han^13,15,23–26^				Combined European^9,18,19,27^				
Locus	Mutation events	Number of meiosis	Mutation rate × 10^−3^(95%CI × 10^−3^)	Mutation events	Number of meiosis	Mutation rate(× 10^−3^)	*P* value	Significance
DYS627	72	4771	15.1(11.8–19.0)	34	2687	12.5(8.8–17.6)	0.4167	
DYS570	31	4771	6.5(4.4–9.2)	31	2530	12.2(8.3–17.4)	0.0151	*
DYS526b	40	3605	11.1(7.9–15.1)	34	2685	12.8(8.8–17.7)	0.6366	
DYS518	47	4771	9.9(7.2–13.1)	44	2687	16.4(11.9–21.9)	0.0156	
DYF404S1	50	3611	13.8(10.3–18.2)	29	2677	11(7.3–15.5)	0.3049	
DYF387S1	49	4771	10.3(7.6–13.6)	41	2687	15.4(11.1–21)	0.061	
DYS626	27	3611	7.5(4.9–10.9)	26	2529	10.4(6.7–15)	0.2634	
DYF399S1	198	3611	54.8(47.6–62.8)	211	2687	78.4(68.6–89.3)	0.0002	***
DYS449	46	4771	9.6(7.1–12.8)	38	2687	14.1(10–19.4)	0.0863	
DYS547	43	3611	11.9(8.6–16.0)	52	2686	19.3(14.5–25.3)	0.021	*
DYS576	59	4771	12.4(9.4–15.9)	36	2687	13.3(9.4–18.5)	0.7472	
DYS612	27	3611	7.5(4.5–10.9)	40	2687	14.8(10.7–20.2)	0.0059	**
DYF403S1a	95	3611	26.3(21.3–32.1)	82	2687	30.7(24.3–37.7)	0.3176	
DYF403S1b	25	3611	6.9(4.5–10.2)	33	2686	12.1(8.5–17.2)	0.0324	*

**Figure 2:**
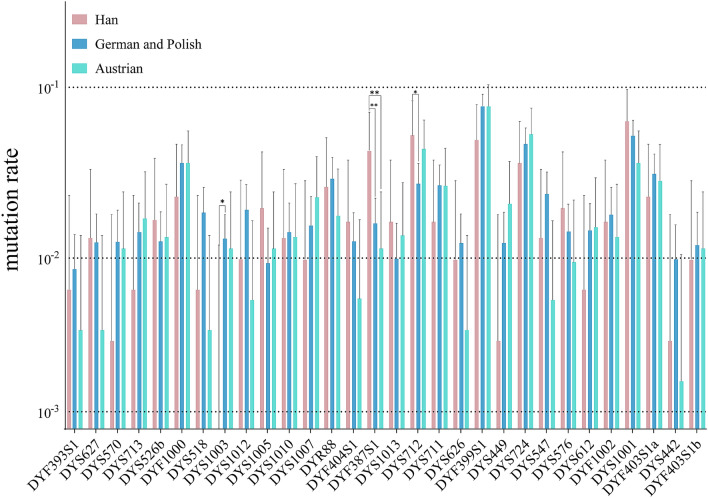
Mutation rate comparison between the Chinese and the European populations presented by locus. Error bars represented the 95% CI. Reference mutation data were acquired from Ballantyne et al^9^, Ralf et al^17^, and Neuhuber et al^19^.

For the 30 Y-STR loci, Fisher's exact test was performed for each pair of mutation events per locus between our studied group and European reference populations, as well (Fig. 2 and Supplementary Table S2)^9,17,19^. The dashed lines in Fig. 2 represent the different mutation rates (10^–1^, 10^–2^, and 10^–3^ per meiosis). In Chinese population, 10 RM Y-STRs showed a relatively lower mutation rate (< 10^–2^ per meiosis, DYS570, DYS713, DYS518, DYS1003, DYS1012, DYS1007, DYS626, DYS449, DYS612, DYF403S1b) and 2 FM Y-STRs (DYS1005 and DYS1013) showed a higher mutation rate (> 10^–2^ per meiosis). Nine loci exhibited several-folds differences between the two populations (DYS570, DYS713, DYS518, DYS1003, DYS1005, DYF387S1, DYS449, DYS612, and DYS442). Significant P-values were spotted at three loci between Chinese population and German and Polish populations, DYS1003 (P-value 0.0370), DYF387S1 (*P* value 0.0058) and DYS712 (*P* value 0.0301), where the mutation rate of the last two loci in our study group was significantly higher. DYF387S1 was also found to have significantly higher mutation rate the Chinese population than in Austrian (*P* value 0.0064). According to the previous mutation studies on DYF387S1, populations from central and southeastern China tend to show lower mutation rates^13,23–26^(0–10.27 × 10^–3^ per meiosis, Supplementary Table S5) than the European populations (11.32–21.50 × 10^–3^ per meiosis)^9,17–19,27^, and went higher in South Asians such as Pakistan (23.0–37.0 × 10^–3^ per meiosis)^10,28^. Inconsistency in mutation rates within an ethnic group was also reported in the Pakistan group, which, like the Han Chinese, is also a largescale population^10,28^. However, the mutation rates for DYS449 and DYS570 were relatively low, consistent with previous findings in Chinese populations^20,23,24^ and lower than in the European populations^9,17^.

According to the nomenclature rules proposed by references^9,17,18^, the 30 Y-STR loci in the Chinese population were re-categorized into 18 RM Y-STRs, 8 FM Y-STR loci, 3 MM Y-STRs, and one locus with no mutation events (mutation rate < 3.26 × 10^–3^ per meiosis) based on the estimated mutation rates. The wide range of intergroup differences observed in the comparison is striking. The inter-population difference in mutation rate has been reported in many other papers^24,25^. A comparative study of SNPs in 1000 genomic data showed an abundance of gene conversions in a particular population, which confirms differences in mutagenesis in different populations^29^. Another study on the differences in Y-STR mutation rates between different haplogroups also demonstrated inter-population differences in mutation rates^12^. The currently endorsed explanation for inter-population mutagenic differences is associated with the uneven distribution of the alleles in different populations^12,30^. For further examine into this hypothesis in future researches, we provided allele frequencies for 30 Y-STR loci in the Chinese population (Supplementary Tables S3 and S4). In order to elucidate population differences in mutation rates, more data from populations with different genetic backgrounds are needed. It is recommended that special care should be taken when applying reference mutation rate data in populations without validation.

### Father-son differentiation rate by the RMplex

**Figure 3:**
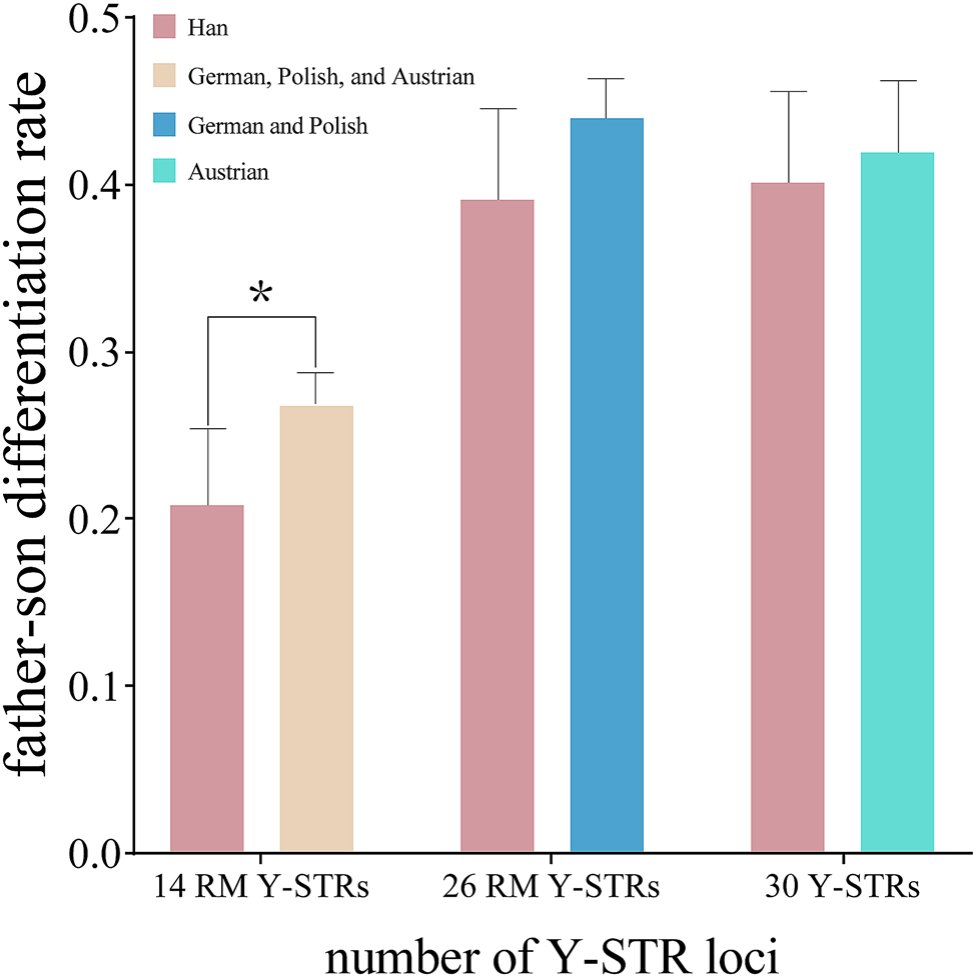
Father-son differentiation rates under different numbers of RM Y-STR loci in Chinese and European populations. Error bars represented 95% CI.

Father-son separation rates were extracted from the previous paper and merged with our data to assess the impact of the newly proposed loci in different populations^17,19^. The visualization of the data is in Fig. 3. The differentiation rates for 14 RM Y-STRs for European were combined from mutation data of German, Polish^17^, and Austrian populations^19^, while the rates for 26 RM Y-STRs and 30 Y-STRs rates were extracted from German and Polish data^17^ and Austrian data^19^, respectively. With the increase in the number of applied RM Y-STR loci, the father-son differentiation rate improved significantly in both populations. However, in all three Y-STR loci sets, the Chinese population had lower father-son pair differentiation than the European populations. Fisher's exact test was applied to evaluate the differences in father-son pair differentiation rates between populations based on the mutation events retrieved from the publications. When tested with 14 RM Y-STR loci, the separation rate was found to be significantly higher in the European population (*P* value 0.0260). When the number of tested loci increased, the differences between populations became non-significant (*P* > 0.05). RM Y-STRs showed strong potential to discriminate father-son pairs in both European and Chinese populations and were able to narrow population differences in distinguishing father-son pairs when tested with a larger number of loci. In conclusion, RM Y-STRs proved to be particularly useful in distinguishing father-son pairs and could be a promising tool for forensic male relative differentiation.

## Methods and materials

### Sample collection and DNA extraction

Buccal swabs of 588 Chinese Han males comprising 281 fathers and 307 sons were collected after receiving written consent, which were then stored at room temperature until extraction^31^. For the case of a father corresponding to more than one son, each father-son pair was treated as an independent pair, reaching a total count of 307 father-son pairs in this study. For each father-son pair, parentage was confirmed using an autosomal STRs EX22 amplification kit (AGCU ScienTech, Wuxi, China). The determination of the paternity is the precondition for the mutation rate analysis. Genomic DNA was extracted using the standard Chelex-100 method and stored at − 20 °C before amplification by polymerase chain reaction (PCR)^32^. This study was approved by the Ethics Committee of the Institute of Forensic Medicine, Sichuan University (approval number: K2019018). All experiments were performed following the relevant guidelines and regulations.

### Y-STRs typing using the RMplex

DNA from the 588 samples was amplified using the primer sequences provided in the previous study^18^. The concentration of each primer pair was in accordance with the previous study, as well. PCR was performed in total volume of 5 μL which consisted of 2.5 μL Multiplex Master Mix (Qiagen, Venlo, Netherlands), 0.8 μL PCR primer mix, 1 ng DNA, and sterilized distilled water (Qiagen, Venlo, Netherlands). PCR was conducted using a ProFlex 96-well PCR System (Thermo Fisher Scientific, Waltham, Massachusetts, U.S.). Thermal cycling protocol was in line with the former paper as well. The amplification products were analyzed using an Applied Biosystems 3500 Genetic Analyzer (Applied Biosystems, Waltham, MA, USA). For accurate genotyping of samples, we used the control DNA 2800 M (Thermo Fisher Scientific, Waltham, Massachusetts, U.S.) as a calibrator, whose genotype was confirmed in the reference paper^18^. Based on the positioning of its peaks in each electrophoresis, we set up the automated genotype bin on the 3500 platform to help obtain the genotypes of the samples. For intermediate alleles, re-amplifications were conducted to confirm the genotype. The Y-STRs haplotype data were then analyzed using GeneMapper™ ID X software (v. 1.5; Applied Biosystems, Waltham, MA, USA). Other conditions were the same as described in the original study^18^. Haplotypes of 123 mutated father-son pairs is shown in Supplementary Table S1.

### Data analysis

The mutation rate per locus was calculated as the ratio between the number of mutation events and the total number of alleles transferred from father to son. For multi-copy loci, peak height ratio differences were not interpreted as mutations. The father-son differentiation rate was calculated by dividing the number of father-son pairs with mutations by the total number of father-son pairs. Calculations were performed manually using Microsoft Excel (One Microsoft Way Redmond, Washington, U.S.), and detailed results are shown in Table 1. 95% Confidence Intervals (95% CI) for mutation rates and father-son differentiation rates were calculated with Clopper-Pearson intervals using a binomial distribution (https://statpages.info/confint.html). Significance of mutation rate per Y-STR and father-son differentiation rate between populations was calculated using Fisher's exact test. ANOVA, linear regression analysis and histograms were performed on GraphPad Prism 8 (www.graphpad.com, Figs. 1, 2, 3). Haplotypes with missing data were discarded in the calculation of mutation rates, differentiation rates and P values. Based on the haplotypes of 281 unrelated fathers from the studied samples, allele frequencies of single-copy and multi-copy loci were calculated and shown in Supplementary Table S3 and S4, respectively.

### Quality control

This study was carried out in a laboratory accredited by the China National Accreditation Service for Conformity Assessment and compliant with ISO 17025. During amplifying and typing of samples, control DNA 2800 M (Thermo Fisher Scientific, Waltham, Massachusetts, U.S.) and sterilized distilled water (Qiagen, Venlo, Netherlands) were used as the positive and negative controls, respectively.

### Ethical approval

All procedures performed in studies involving human participants followed the ethical standards of the Ethics Committee of the Institute of Forensic Medicine, Sichuan University (K2019018), and complied with the 1964 Declaration of Helsinki and its later amendments or comparable ethical standards. Informed consent was obtained from all participants and/or their legal guardians included in the study.

## Conclusion

In this study, we estimated the mutation rate and paternal differentiation rate of 26 RM Y-STRs and 4 FM Y-STRs in 307 Chinese father-son pairs for the first time, using the newly introduced RMplex panel. Based on the estimated data, we also compared the mutation rates in Chinese and European reference populations for the first time. For the previously reported 14 RM Y-STR loci, mutation rates of former studies on Chinese and European populations were pooled together and recalculated, respectively. The highest meiosis number reached 4771 and 2687 in the two pooled groups, respectively. Five loci were found to have significant differences between the populations (DYS570, DYS399S1, DYS547, DYS612, and DYF403S1b). The broad differences observed indicated the non-negligible discrepancy in the mutagenesis between the inter-continental populations.

In our study on 30 Y-STR loci using the RMplex, the estimated mutation rates in the Chinese population showed extensive differences compared to the European populations, as well. Nine loci were several-fold different in the two populations, with two loci having significantly higher mutation rates in the Chinese population (*P* < 0.05). Based on the naming rules proposed in previous studies, 18 loci could be re-categorized into 18 RM Y-STRs, 8 FM Y-STRs, 3 loci MM Y-STRs, and one locus with no mutation event in the Chinese population. With these rapidly mutating loci, significant differences in mutations among different continental populations were identified. With the RMplex panel, 40.06% of father-son pairs were distinguished, compared to only 20.84% using the original 14 RM Y-STRs, indicating that RMplex is beneficial for analyzing paternally related males. A significant decrease in differentiation rate was observed in the Chinese population when tested with the originally identified 14 RM Y-STR (*P* < 0.05), compared with reference European populations. When tested with more Y-STR loci, inter-population differences became non-significant, suggesting the compatibility of the novel RM Y-STR loci in both populations. In general, RMplex showed extensive variabilities across populations and has a strong potential to discriminate father-son pairs. After further validation in populations with different genetic backgrounds, RMplex could be a promising tool for forensic male relative differentiation.

## Supplementary Information

Supplementary Information 1.

Supplementary Information 2.

Supplementary Information 3.

## Data Availability

Data included in this manuscript has been uploaded to YHRD (accession number YA004395) and is also available at Supplementary Table.
